# The Effect of Sodium-Dependent Glucose Cotransporter 2 Inhibitor Tofogliflozin on Neurovascular Coupling in the Retina in Type 2 Diabetic Mice

**DOI:** 10.3390/ijms23031362

**Published:** 2022-01-25

**Authors:** Junya Hanaguri, Harumasa Yokota, Akifumi Kushiyama, Sakura Kushiyama, Masahisa Watanabe, Satoru Yamagami, Taiji Nagaoka

**Affiliations:** 1 Division of Ophthalmology, Department of Visual Science, Nihon University School of Medicine, Itabashi, Tokyo 173-8601, Japan; locksteady@icloud.com (J.H.); atokoy18@gmail.com (H.Y.); masahisa_watanabe12-1@yahoo.co.jp (M.W.); yamagami.satoru@nihon-u.ac.jp (S.Y.); 2 Department of Pharmacotherapy, Meiji Pharmaceutical University, Kiyose, Tokyo 204-8588, Japan; kushiyama@my-pharm.ac.jp; 3 Division of Life Science, Department of Nursing, National College of Nursing, Kiyose, Tokyo 204-8575, Japan; kushiyamake@gmail.com

**Keywords:** diabetic retinopathy, neurovascular coupling, SGLT2 inhibitor, glial cell function, microvascular complication

## Abstract

We investigated the effect of tofogliflozin, a sodium-dependent glucose cotransporter 2 inhibitor (SGLT2i), on retinal blood flow dysregulation, neural retinal dysfunction, and the impaired neurovascular coupling in type 2 diabetic mice. Tofogliflozin was added to mouse chow to deliver 5 mg/kg/day and 6-week-old mice were fed for 8 weeks. The longitudinal changes in the retinal neuronal function and blood flow responses to systemic hyperoxia and flicker stimulation were evaluated every 2 weeks in diabetic db/db mice that received tofogliflozin (*n* =6) or placebo (*n* = 6) from 8 to 14 weeks of age. We also evaluated glial activation and vascular endothelial growth factor (VEGF) expression by immunofluorescence. Tofogliflozin treatment caused a sustained decrease in blood glucose in db/db mice from 8 weeks of the treatment. In tofogliflozin-treated db/db mice, both responses improved from 8 to 14 weeks of age, compared with vehicle-treated diabetic mice. Subsequently, the electroretinography implicit time for the oscillatory potential was significantly improved in SGLT2i-treated db/db mice. The systemic tofogliflozin treatment prevented the activation of glial fibrillary acidic protein and VEGF protein expression, as detected by immunofluorescence. Our results suggest that glycemic control with tofogliflozin significantly improved the impaired retinal neurovascular coupling in type 2 diabetic mice with the inhibition of retinal glial activation.

## 1. Introduction

Diabetic retinopathy continues to be a leading cause of blindness [1]. The prospective observational clinical study with type 2 diabetes revealed that hemoglobin A1c was the only factor associated with both the incidence and progression of diabetic retinopathy (DR) [2], suggesting that blood glucose levels are very important to prevent the DR in type 2 diabetes. Therefore, glycemic control is the most important goal after the onset of diabetes with no apparent retinopathy.

Sodium-glucose cotransporter 2 (SGLT2) inhibitors (SGLT2i) are a new class of oral anti-diabetic agent that reduces the blood glucose level by inhibiting glucose reabsorption in the proximal renal tubules. These agents are commonly used by young obese patients with type 2 diabetes because weight reduction can be expected as calories are lost when glucose is excreted in the urine [3]. Recently, some clinical case series have shown the beneficial effects of SGLT2i on DR [4,5] and diabetic macular edema [6,7]. However, the EMPA-REG outcome trial could not identify a beneficial effect of empagliflozin on DR [8]. Moreover, no study has examined the effect of SGLT2i on retinal function in diabetic animals.

Our recent study revealed that retinal blood flow (RBF) does not respond to systemic hyperoxia and flicker stimulations in db/db mice with early type 2 diabetes, and no apparent changes in resting retinal perfusion were observed [9]. These results suggest that long-term glucotoxicity is a central mechanism for impaired retinal neurovascular coupling in type 2 diabetic mice. Glucose lowering by the early detection of RBF dysregulation and prompt treatment may, therefore, protect retinal tissue and prevent or slow the development of irreversible retinopathy. We investigated the effect of long-term (8 weeks) systemic treatment with tofogliflozin, an SGLT2 inhibitor, on RBF dysregulation and neural retinal dysfunction in type 2 diabetic mice.

## 2. Results

### 2.1. The Effect of the Longitudinal Administration of Tofogliflozin on Glycemic Control

In this study, the drug treatment of db/db mice was initiated from 6 weeks of age. Blood glucose was measured every 2 weeks from 6 weeks before the start of treatment to 14 weeks of age. The casual blood glucose levels in db/db mice treated with tofogliflozin (*n* = 6) significantly decreased in comparison with untreated db/db mice (*n* = 6) from 8 to 14 weeks. At 14 weeks of age, the blood glucose levels were untreated and SGLT2i-treated db/db mice were 440.2 ± 54.0 and 262.2 ± 43.3 mg/dL, respectively (Table 1).

### 2.2. Longitudinal Changes of Systemic and Ocular Parameters

**Figure 1:**
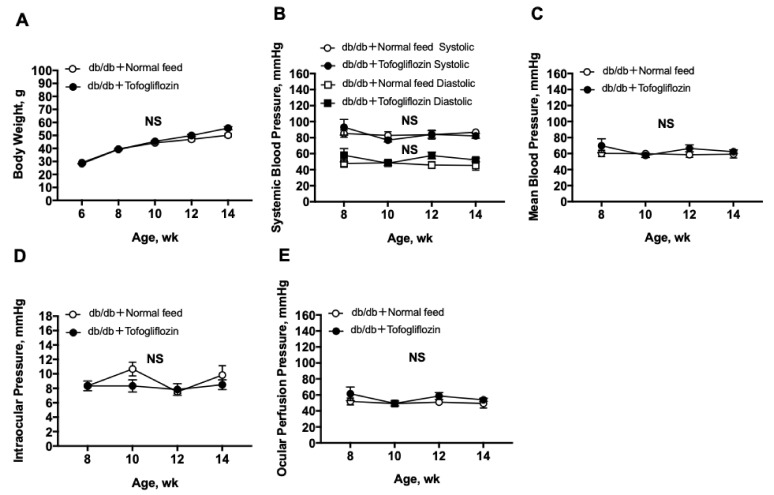
Tofogliflozin did not change the level of other systemic or ocular parameters: (**A**) body weight; (**B**) systemic BP; (**C**) mean BP; (**D**) IOP; (**E**) OPP. Body weight was measured every 2 weeks from 8 weeks to 14 weeks of age. Systemic BP, mean BP, IOP, and OPP were measured every 2 weeks from 8 weeks to 14 weeks of age. Each parameter was compared between tofogliflozin-treated db/db mice (db/db + tofogliflozin) (*n* = 6) in db/db mice fed normal chow (db/db + normal feed) (*n* = 6) by two-way repeated-measures analysis of variance (ANOVA). Data are expressed as the mean ± SEM. NS indicates no significant difference between groups.

During the experiment, there were no significant differences in body weight, systemic blood pressure (BP), intraocular pressure (IOP), or ocular perfusion pressure (OPP) between treated and untreated mice (Figure 1).

### 2.3. Longitudinal Assessment of Resting RBF in Diabetic Mice

**Figure 2:**
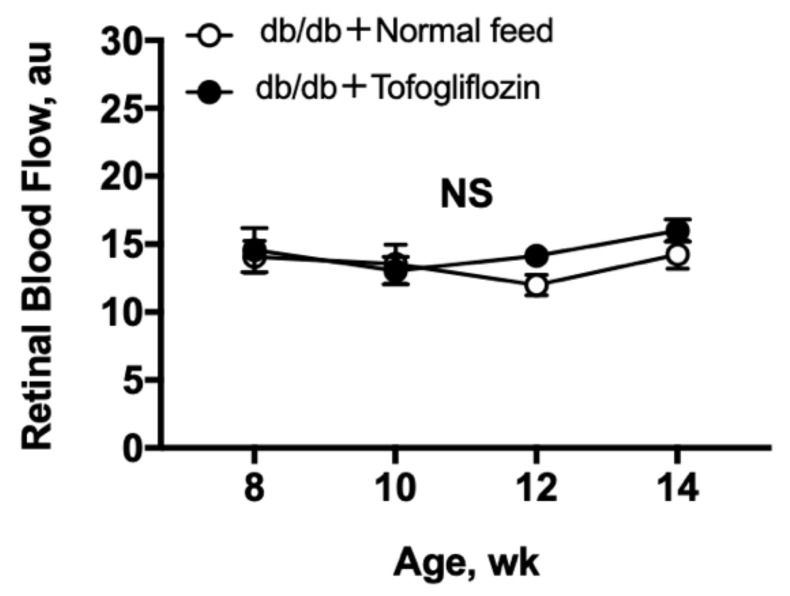
Longitudinal resting RBF remains stable throughout the measurement period from 8 to 14 weeks of age in db/db mice and were not different for db/db mice treated with tofogliflozin (*n* = 6) and db/db mice fed normal chow (*n* = 6) from 8 to 14 weeks of age. RBF was measured every 2 weeks, and the statistical analyses of the results of each treatment over time were performed by a one-way repeated measures ANOVA (*p* = 0.32 for db/db + normal feed mice and *p* = 0.34 for db/db + tofogliflozin mice). The differences between 2 weeks in the resting RBF were tested in the 2 groups by a two-way repeated-measures ANOVA (*p* = 0.24). au indicates arbitrary unit; NS indicates no significant difference between groups and within a group. Data are expressed as the mean ± SEM.

The resting RBF is shown in Figure 2. Both untreated db/db and treated db/db showed a steady resting RBF from 8 to 14 weeks of age, with no significant difference between the groups (two-way repeated-measures analysis of variance (ANOVA)).

### 2.4. Longitudinal Assessment of RBF in Response to Systemic Hyperoxia in Diabetic Mice

**Figure 3:**
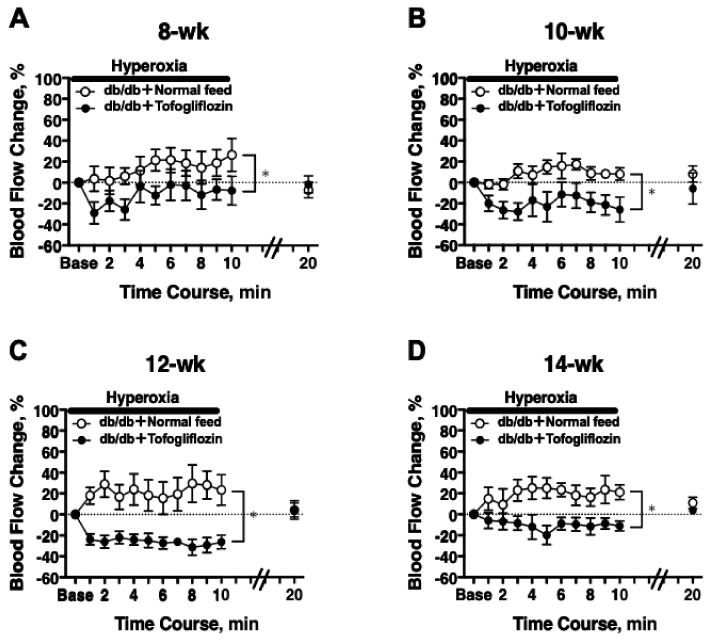
The RBF response to systemic hyperoxia was longitudinally retained in db/db mice treated with tofogliflozin (db/db + tofogliflozin, *n* = 6) and blunted in db/db mice fed normal chow (db/db + normal feed, *n* = 6) from 8 to 14 weeks of age: (**A**) 8 weeks of age; (**B**) 10 weeks of age; (**C**) 12 weeks of age; (**D**) 14 weeks of age. * *p* < 0.05 between groups analyzed by repeated-measures ANOVA; solid bar = period of hyperoxia.

In 8-week-old db/db mice, a significant difference in the hyperoxia-induced change in RBF was observed between the treatment groups (two-way repeated-measures ANOVA; Figure 3A). The significant differences in hyperoxia-induced flow also were observed at 10, 12, and 14 weeks of age (Figure 3B–D).

### 2.5. Longitudinal Assessment of RBF in Response to Flicker Stimulation in Diabetic Mice

**Figure 4:**
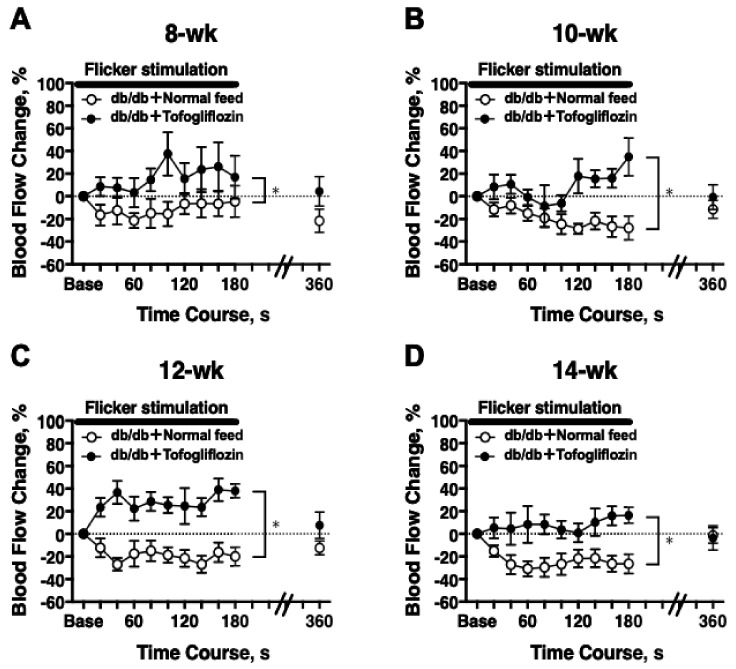
The RBF response to flicker stimulation was longitudinally retained in db/db mice treated with tofogliflozin (db/db + tofogliflozin, *n* = 6) and blunted in db/db mice fed normal chow (db/db + normal feed, *n* = 6) from 8 to 14 weeks of age: (**A**) 8 weeks of age; (**B**) 10 weeks of age; (**C**) 12 weeks of age; (**D**) 14 weeks of age. * *p* < 0.05 between groups analyzed by a repeated-measures ANOVA; solid bar = period of flicker stimulation.

The temporal change in RBF in response to a 3 min light flicker stimulation was evaluated on experimental day 2. In 8-week-old db/db mice, there was a significant difference in the flicker-induced change in RBF between untreated and treated mice (two-way repeated-measures ANOVA; Figure 4A). Significant differences in flicker-induced flow changes also were at 10, 12, and 14 weeks of age (Figure 4B–D).

### 2.6. Longitudinal Assessment of Electroretinography (ERG) Parameters

**Figure 5:**
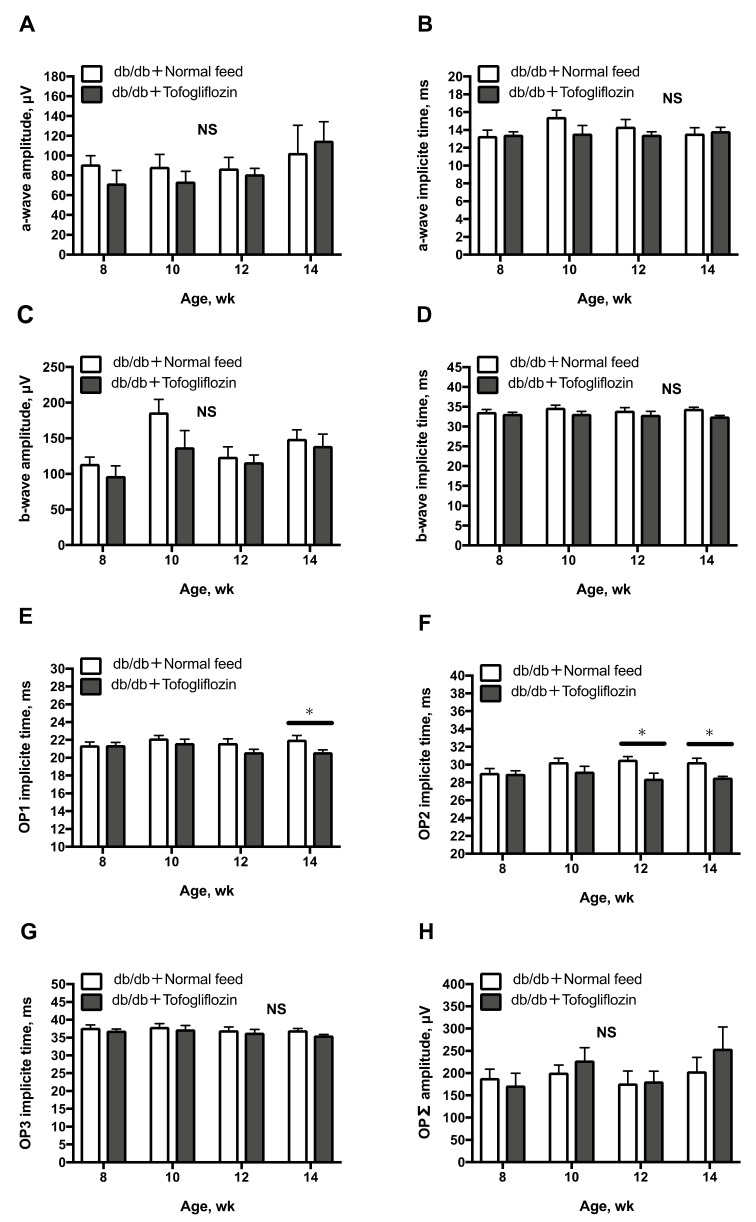
Longitudinal assessment of ERGs in db/db mice treated with tofogliflozin (db/db + tofogliflozin, *n* = 6) and blunted in db/db mice fed normal chow (db/db + normal feed, *n* = 6) from 8 to 14 weeks of age: (**A**) amplitude of the a-wave; (**B**) implicit time of the a-wave; (**C**) amplitude of the b-wave; (**D**) implicit time of the b-wave; (**E**) implicit time of OP1 waves; (**F**) implicit time of OP2 waves; (**G**) implicit time of OP3 waves; (**H**) total amplitudes of OP waves (ΣOP). There were no significant differences in the implicit time or amplitude of the a-waves (**A**,**B**) and b-waves (**C**,**D**) between the db/db + tofogliflozin mice and db/db + normal feed mice. There were no significant changes in the implicit time of the OP3 (**G**) waves or the total amplitudes of the OP waves (ΣOP) (**H**) between the groups of mice (two-way repeated-measures ANOVA). The implicit time of OP1 (**E**) increased significantly in db/db + normal feed mice at 14 weeks of age, and the implicit time of the OP2 waves (**F**) increased significantly in db/db + normal feed mice from 12 to 14 weeks of age (two-way repeated-measures ANOVA). * *p* < 0.05, between groups.

The amplitude or implicit time of the a-wave and b-wave on ERG in treated and untreated db/db mice showed no significant changes with age (Figure 5A–D; one-way and two-way repeated-measures ANOVA). While no significant differences were found between treated and untreated db/db mice in the implicit time of OP3 (Figure 5G) and the ΣOP amplitude (Figure 5H), the implicit times of OP1 and OP2 in SGLT2i tofogliflozin-treated db/db mice decreased significantly, compared with those in untreated db/db mice from 12 to 14 weeks of age (Figure 5E; two-way repeated-measures ANOVA).

### 2.7. Comparison of the Maximal Change in RBF in Response to Hyperoxia and Flicker Stimulation at 14 Weeks of Age in Diabetic Mice and db/m Non-Diabetic Control Mice

**Figure 6:**
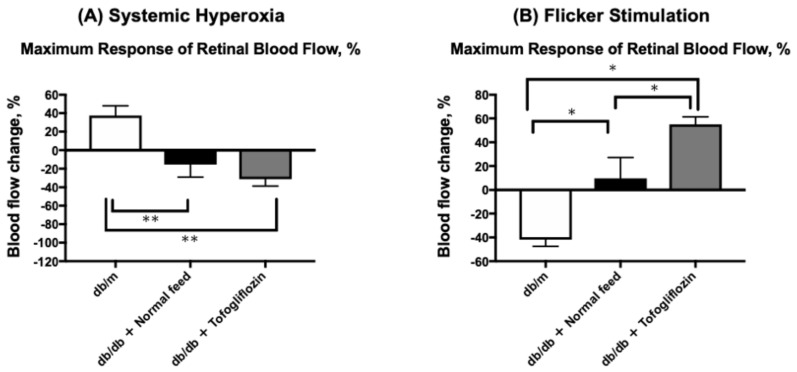
Maximal changes in RBF from baseline in response to hyperoxia (**A**) and flicker stimulation (**B**) in 14-week-old mice of db/m (*n* = 5) as a non-diabetic control and untreated db/db mice (*n* = 6) and tofogliflozin-treated diabetic mice (*n* = 6). * *p* < 0.05, ** *p* < 0.01 compared with db/m by a one-way ANOVA followed by Holm-Sidak test.

At 14 weeks of age, the same protocol for systemic hyperoxia and flicker stimulation was performed in two diabetic mice and db/m control mice groups (Figure 6). We previously confirmed that the RBF response to both stimulations remained stable in db/m non-diabetic control mice from 8 to 20 weeks of age [9]. RBF measurements were performed only once at only 14 weeks of age in line with animal welfare criteria for our institution. Figure 6 shows the changes in RBF in response to both systemic hyperoxia (Figure 6A) and flicker stimulation (Figure 6B). In Figure 6, the changes observed in control db/m mice were not present in the untreated db/db mice; however, in the SGLT2i tofogliflozin group, these changes were approximately half of those in the db/m control group.

### 2.8. Beneficial Effect of the Systemic Administration of Tofogliflozin on Glial Fibrillary Acidic Protein (GFAP) and VEGF Expression

**Figure 7:**
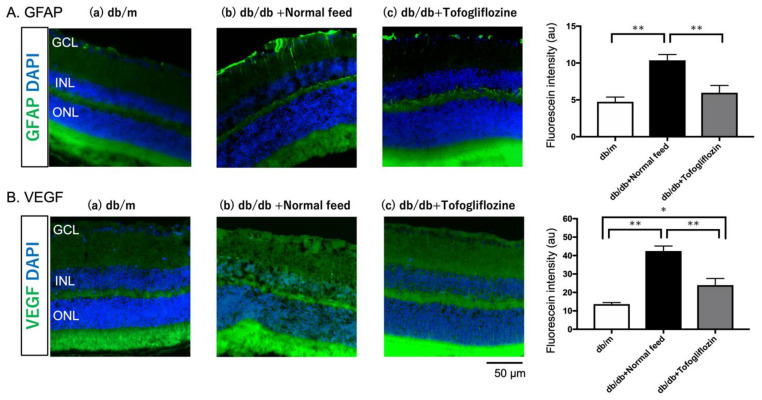
Effect of SGLT2i tofogliflozin on the glial activation (**A**) and production of VEGF (**B**). Comparison of GFAP immunofluorescence (green) between representative samples from in db/m (**a**) and untreated (**b**) or SGLT2i-treated (**c**) db/db mice at 14 weeks. In the diabetic retina with normal feed (**b**), the end-feet of the Müller cells show abundant GFAP immunofluorescence, and the radial processes are stained intensely throughout both the inner and outer retina. In the diabetic retina with tofogliflozin (**b**), the immunofluorescence of both GFAP and VEGF was reduced and comparable with that in db/m control mice (**a**). There were significant differences in the fluorescein intensities of GFAP (*p* = 0.005) and VEGF (*p* < 0.0001) among the three groups by one-way ANOVA. There were significant increases in intensities of GFAP and VEGF in the untreated db/db mice (*n* = 6), compared with the non-diabetic db/m mice (*n* = 5). These increases were significantly ameliorated in the tofogliflozin-treated murine retina (*n* = 6). * *p* < 0.05, ** *p* < 0.01. Nuclei were labeled with DAPI (blue). ONL, outer nuclear layer; INL, inner nuclear layer; GCL, ganglion cell layer. Scale bar = 50 μm.

To clarify the beneficial effect of tofogliflozin on the pathogenesis of DR, we performed immunohistochemical analysis to investigate whether the long-term systemic administration of SGLT2i ameliorates the glial activation and VEGF expression in the retina of the type 2 diabetic murine model. As shown in Figure 7, tofogliflozin decreased the overexpression of GFAP (Figure 7A) and VEGF (Figure 7B) in the db/db mouse retina. There were significant increases in fluorescein intensities of GFAP and VEGF in the untreated db/db mice, compared with non-diabetic db/m mice, but the increased intensities of both GFAP (Figure 7A) and VEGF (Figure 7B) were significantly reduced in SGLT2i-treated mice retina.

## 3. Discussion

In the current study, we confirmed that the long-term administration of SGLT2i tofogliflozin decreased the blood glucose level, compared with untreated db/db mice (Table 1). Persistent hyperglycemia drives complex biochemical and molecular changes that lead to oxidative stress/inflammation, vascular endothelial cell damage, capillary ischemia, and tissue hypoxia [10]. Indeed, we recently confirmed that RBF dysregulation in response to flicker stimulation, which indicates impaired neurovascular coupling in the retina [11], occurred from 8 to 20 weeks of age in db/db mice before the apparent neuronal abnormalities, which can be detected by ERG [9]. As pericyte loss was observed at 24 weeks of age but not 12 weeks of age in db/db mice [12], the impaired neurovascular coupling in the retina may be the earliest event in the diabetic retina. In the current study, glycemic control by the SGLT2i tofogliflozin ameliorated the impaired blood flow response to flicker stimulation with the improvement of glycemic control, suggesting that glucotoxicity to the retinal neurovascular unit can be a primary risk factor for impaired neurovascular coupling in the retina. Our findings seem to support the recent American Diabetic Association statement that DR should be reclassified from a microvascular complication to a neurovascular complication since damage to the specialized neurons in the eye plays an important role in the development and progression of DR [13].

Previous clinical studies have shown that the SGLT2i treatment decreases systemic BP [14,15]. However, there were no significant changes in systemic BP, IOP, or OPP in either untreated or SGLT2i-treated mice during the follow-up period in the current study (Figure 1). In contrast, a previous animal study also reported that empagliflozin ameliorates kidney injury in type 2 diabetic female mice by promoting glycosuria without any changes in systemic BP [16]. Their findings seem to be comparable to ours.

We used the SGLT2 inhibitor tofogliflozin to restore glycemic control in db/db mice. SGLT2 inhibitors are the latest class of anti-diabetic medications. In addition, a recent clinical study reported that another SGLT2i, empagliflozin, reduces the risk of major adverse cardiovascular events [17] and reduces the progression of kidney diseases [18], suggesting that SGLT2is may have a beneficial effect on both macrovascular and microvascular complications in diabetic patients. Indeed, a recent real-world cohort study showed that SGLT2is may be associated with a lower risk of DR, compared with DPP4is [5]. In addition, the treatment of type 2 diabetic patients with an SGLT2i slowed the progression of DR in comparison with sulfonylurea, which occurs independently of its effect on glycemic control [4]. Taken together, a large-scale prospective clinical study to examine the effect of SGLT2i on the prevention of DR is warranted.

Eid et al., reported that the long-term administration of empagliflozin for 10 weeks from 6 to 16 weeks of age did not ameliorate any microvascular complications in db/db mice [19]. In their study, DR was assessed by the measurement of cleaved apoptotic DNA in the retina using an ELISA. We confirmed that the RBF abnormalities occurred at 8 weeks of age in db/db mice, which preceded the retinal neuronal dysfunction evaluated by ERG. Although different types of SGLT2is were used in the two studies, RBF provocation of systemic hyperoxia and flicker stimulation may be useful as a biomarker for the detection of early retinal dysfunction.

A previous study showed that sodium-dependent glucose uptake is present in bovine retinal pericytes and human retinal endothelial cells [20]. In contrast, histologic studies assessing the expression of SGLT2 directly in human retinas have not been reported widely in the literature [21]. In our preliminary study, we could not find any expression of SGLT2 in the db/db mouse retina in Supplementary Materials (Figure S1). Therefore, we speculate that the glucose-lowering effect induced by the SGLT2i tofogliflozin may ameliorate the diabetes-induced impairment of RBF regulation in response to flicker stimulation and systemic hyperoxia. If this is the case, the improvement in the neurovascular function in the retina of db/db mice may be associated with glycemic control rather than the inhibition of SGLT2 in the retina.

We found that the long-term administration of tofogliflozin decreased the GFAP expression in the db/db mouse retina (Figure 7). Our result seems to be comparable with an increasing number of studies that show that diabetes is associated with reactive Müller cell gliosis [22,23], which is characterized by increased levels of GFAP. As the retinal glial cells play important roles in the regulation of RBF in response to systemic hyperoxia [24] and flicker stimulation [25], the beneficial effect of SGLT2i on retinal glial cells may contribute to the recovery of the diabetes-induced retinal dysfunction via improved glycemic control. In the diabetic brain, the SGLT2i empagliflozin prevented glial activation in the neurovascular unit, suggesting that empagliflozin may provide neuroprotection in the diabetic brain via the protection of glial cells [26]. In addition, the non-selective SGLT inhibitor phlorizin ameliorates the endothelial dysfunction link with the activation of the PI3K/AKT/eNOS signaling pathway and augmentation of the release of nitric oxide (NO) [27], and SGLT2i EMPA, and DAPA restores NO bioavailability by inhibiting reactive oxygen species (ROS) generation [28]. It is difficult to confirm the exact mechanism of neurovascular coupling in the retina in response to flicker stimulation; glial activation and/or the restored impaired endothelial function may be involved in the restoration of RBF regulation in response to the systemic hyperoxia and flicker stimulation.

In the present study, we first observed that the increased VEGF expression in the retina decreased with long-term SGLT2i treatment in diabetic mice at 14 weeks of age (Figure 7). Amin et al. [29] revealed that VEGF immunopositivity was present in eyes with no retinal vascular anatomic abnormality in retinal digest preparations from diabetic patients with non-proliferative DR. Their results are comparable to our findings. Wang et al., clearly showed that the conditional VEGF knockout (KO) in Müller cells of diabetic mice exhibited significantly reduced leukostasis, expression of inflammatory biomarkers, and vascular leakage, compared with STZ-induced diabetic control mice. [30] These results suggest that the glucotoxicity-related VEGF overexpression may be produced from the activated Müller cells in the diabetic retina [29]. In addition, Mu et al., reported that the levels of VEGF mRNA and protein increased in cultured Müller cells under high glucose concentrations [31]. As persistent gliosis leads to the loss of normal Müller glial cell function and subsequent loss of retinal neurons [32], our data suggest that glucotoxicity-induced gliosis of Müller cells, which can be detected the increased GFAP expression, may lead to the overproduction of VEGF in the diabetic retina as an early event in the diabetic retina. Moreover, we believe that the long-term administration of the SGLT2i can ameliorate the retinal neurovascular coupling by reducing the activation of Müller cells and the overexpression of VEGF by lowering blood glucose levels.

Clermont et al., reported that acute glycemic control by primary intervention using insulin pumps caused the normalization of the RBF in type 1 STZ-induced diabetic rats [33], suggesting that glucotoxicity may be involved with the impaired RBF in diabetic animals. However, no studies have investigated whether long-term glycemic control ameliorates the RBF dysregulation in diabetic animals. In the present study, we reported, for the first time, that long-term glycemic control by SGLT2i results in the improvement of the RBF response to flicker stimulation and systemic hyperoxia in type 2 diabetic mice from 8 to 14 weeks of age. In addition, a long-term glycemic control by SGLT2i reduced the delay of the implicit time of the oscillatory potentials from 12 to 14 weeks of age in diabetic mice. The current results suggest that glycemic control is very important for maintaining a normal retinal neuronal and vascular function in the early stage of diabetes.

Although some recent clinical studies have reported the beneficial effect of SGLT2i on DR [4,5] and diabetic macular edema [6,7], the EMPA-REG outcome trial could not find a beneficial effect of empagliflozin on DR [8]. Eid et al., reported that the long-term administration of empagliflozin for 10 weeks from 6 to 16 weeks of age did not ameliorate any microvascular complications in db/db mice [19]. In their study, they evaluated cleaved apoptotic DNA using an ELISA to assess “DR”. In the current study, we confirmed that retinal functional abnormalities occurred at 8 weeks of age in db/db mice. A clinical trial is needed to investigate whether the SGLT2is may have a beneficial effect on DR, especially at the early stage of DR, by evaluating the abnormalities of the RBF regulation in response to systemic hyperoxia and flicker stimulation, as we conducted in our study. Glycemic control by SGLT2i may contribute to the prevention of diabetic macular edema and DR.

We previously confirmed that the RBF response to systemic hyperoxia and flicker stimulation remains stable from 8 to 20 weeks of age in db/m non-diabetic control mice [9]. Due to the animal welfare criteria in our institution, we performed only one RBF and ERG experiment at 14 weeks of age in db/m mice. We found that the maximal responses of the RBF in response to hyperoxia and flicker stimulation in db/db mice with tofogliflozin had not fully recovered to the same levels as those in non-diabetic db/m mice at 14 weeks of age (Figure 6), but there were no significant differences in either response between the groups, suggesting that tofogliflozin can improve the normal responses of the RBF to systemic hyperoxia and flicker stimulation. Further study is warranted to confirm the effect of supplementation on the prevention of DR in a diabetic animal model, which expresses DR, or in human patients with diabetes.

The present study had some limitations. First, we could not exclude the possibility that a direct effect of the inhibition of SGLT2 in the retina, which was reported in bovine retinal pericytes, may have had some effects on our results in accordance with the glycemic control by the systemic administration of SGLT2i in the current study. Second, we did not confirm whether SGLT2i tofogliflozin prevents the pathological changes of DR. As it has been reported that the pericyte loss occurred at 24 weeks of age in db/db mice [12], a longitudinal observational study with a longer follow-up period is needed.

In conclusion, we found that long-term glycemic control with SGLT2i tofogliflozin significantly ameliorated the glial activation and VEGF expression in the retina and improved the RBF regulation dysfunction and neural retinal dysfunction in type 2 diabetic mice. These results suggest that the SGLT2i may contribute to the prevention of DR and diabetic macular edema.

## 4. Materials and Methods

### 4.1. Animal Preparation

The Nihon University Ethical Committee approved the animal experiments, which were carried out according to the tenets of the Association of Research in Vision and Ophthalmology. We also confirmed that this survey was conducted in accordance with the ARRIVE guidelines (https://arriveguidelines.org (accessed on 13 December 2021)). Five-week-old male C57BL/KsJ-db/db mice (BKS.Cg-Dock7m +/+ Leprdb/J; *n* = 12) and 13-week-old male db/m (non-diabetic congenic littermates, *n* = 5) control mice were obtained from Charles River Laboratories Japan Co., Ltd. (Yokohama, Japan). They were divided into a placebo group (*n* = 6), with ad libitum access to normal chow, and a treatment group (*n* = 6), with ad libitum access to chow containing tofogliflozin hydrate. We obtained tofogliflozin from Kowa Co., Ltd. (Tokyo, Japan). Only male db/db mice were used because diabetes is more severe in male db/db mice than in females [34]. Blood glucose levels were measured from the tail vein (glucose assay kit; Abbott Laboratories, Abbott Park, IL, USA). Mice were housed in a temperature-controlled room, with a 12 h light-dark cycle and ad libitum access to food and water. Throughout the experiment, mice were anesthetized with 2% isoflurane (Pfizer, Tokyo, Japan) continuously inhaled at a flow rate of 1.5 L/min. The pupil was dilated with 0.5% tropicamide (Santen Pharmaceutical Co., Ltd., Osaka, Japan). The rectal temperature was measured and a heated blanket was used to maintain the temperature between 37 and 38 °C.

### 4.2. Chemicals and Systemic Administration Protocol

Tofogliflozin was kindly provided by Kowa Company, Ltd. The db/db mice were randomly allocated to either the untreated group (*n* = 6) or the treatment group (*n* = 6). The db/db mice in both groups had ad libitum access to solidified regular food. In the treatment group, the solidified food was mixed with 0.004% tofogliflozin (5 mg/kg/day) at the same dose as reported in the previous study [35].

### 4.3. Systemic BP and IOP Measurements

Systemic BP and IOP were measured 30 min after the induction of anesthesia. Tail systolic BP (SBP) and diastolic BP (DBP) were measured using an automatic sphygmomanometer (THC-31, Softron, Tokyo, Japan). IOP was measured by a handheld tonometer (TonolabTV02, ME Technical, Tokyo, Japan). The mean arterial pressure (MABP) was derived from the standard formula: MABP = DBP + (SBP − DBP)/3. 

The OPP was calculated using the following formula for an experimental animal in the prone position [36]: OPP = MABP − IOP. 

### 4.4. RBF Measurement

RBF was measured using the LSFG Micro system (Softcare Co., Ltd., Fukutsu, Japan), as previously described [9]. In brief, the LSFG-Micro system includes a standard charge-coupling device camera (700 × 480 pixels) and a diode laser (830 nm wavelength) mounted on a stereomicroscope (SZ61TR, Olympus Corporation, Tokyo, Japan). The mean blur rate (MBR), a relative indicator of blood flow velocity, is generated from the speckle pattern blur formed by the backscattered light of a coherent laser due to the movement of blood cells. The MBR obtained from the area around the optic nerve head (ONH) and its vascular area reflects the entire retinal circulation and is used as an indicator of RBF [36]. In this study, the MBR was obtained as previously described [9]. The mouse was placed on the stand with the left eye facing up. A drop of viscoelastic material was dropped and the cover glass was gently placed on the left cornea. The ONH margin was shown by manually placing a rubber O ring (diameter: 1.37 mm) on the ONH fundus image. The MBR was continuously acquired at 30 frames/second within the O-ring area. The average MBR was analyzed using the LSFG analyzer software program (version 3.2.19.0, Softcare Co., Ltd., Fukutsu, Japan).

### 4.5. Induction of Systemic Hyperoxia

Systemic hyperoxia was induced by the inhalation of 100% oxygen over 10 min, as explained in our previous study [36]. The baseline value was determined as the average of three consecutive flow measurements taken at 1 min intervals for 3 min. To determine the onset of hyperoxia, RBF measurements were taken every minute for 20 min during hyperoxia (10 min of stimulation) and after the end of hyperoxia (10 min of recovery) [9,36].

### 4.6. Flicker Light Stimulation

A 12 Hz flicker stimulation was used because this frequency causes the maximal RBF response in mice, as explained in our previous study [36]. The ambient light was reduced to less than 1 lux before inducing flicker stimulation. As previously reported, mice were dark adapted for 2 h, and the light intensity of flicker stimulation was 30 lux in rod-dominant mouse retinas [9,36]. RBF was measured at 20 s intervals, with both 3 min flickers. Stimulation and recovery lasted for 3 min. Baseline values were calculated using the average of three consecutive flow measurements taken at 1 min (20 s intervals) before the start of light flicker stimulation.

### 4.7. ERG Recording

Before ERG, mice were dark adapted for at least 12 h before being transferred to a room with dim red light. Full-field ERG was recorded using PuREC (Mayo, Inazawa, Japan) under systemic anesthesia with isoflurane. The ground electrode was attached to the tail, the reference electrode was attached to the mouth, and the corneal electrodes were placed on the corneal surface. As previously reported, we achieved maximal responses for both cones and rods using a 3.0 candela/m^2^ flash [36]. OPs are small radio frequency vibration wavelets superimposed on the upper limb of the b-wave. OP wavelets were labeled sequentially as OP1–OP3, starting with the first positive peak detected. The OP amplitude (positive amplitude of the peak-negative amplitude of the peak of the previous peak), and implicit time were measured. The OP amplitude was calculated by adding the first three positive wavelets and expressed as the ΣOP amplitude.

### 4.8. Experimental Protocols

At 6 weeks of age, before the administration of tofogliflozin, we measured the body weight and blood glucose. The following protocol was performed in each animal for 3 consecutive days, every 2 weeks from 8 to 14 weeks of age, for longitudinal assessment of RBF regulation and the neural function: The systemic hyperoxia reaction of RBF occurred on the first day; the following day, systemic BP, IOP, and OPP were not altered by the reaction to high oxygen or light flicker stimulation. On the third day, we recorded the ERG. An independent masked observer (A.K.) performed all data calculations and statistical analysis.

### 4.9. Immunohistochemistry

After the measurement at 14 weeks of age, all animals, including untreated (*n* = 6) and SGLT2i-treated (*n* = 6) db/db and db/m (*n* = 5) mice, were euthanized, and the eyeballs were excised, as follows: Sternotomy was performed under systemic anesthesia with 3% isoflurane; normal saline was perfused into the left ventricle to wash out the circulating blood, immediately, followed by perfusion of 4% paraformaldehyde (PFA), and the eyeballs were enucleated. The eyeballs were stored in 4% PFA at 4 °C overnight; then, after a couple of washings in phosphate-buffered saline, they were embedded in Tissue-Tek OCT Compound (Sakura Finetek Japan, Tokyo, Japan) and stored at −80 °C until analysis.

A total of 10 micrometer-thick sections were prepared with a cryostat (HM505, Microm, Walldorf, Germany). Sections were stained with GFAP (ready to use, Dako, Glostrup, Denmark), ionized calcium-binding adapter molecule 1 (iba-1; dilution, 1:200; Wako Pure Chemical Industries Ltd., Osaka, Japan), and VEGF (1:100, Sigma-Aldrich, St. Louis, MO, USA) overnight at 4 °C. Next, they were incubated with secondary donkey anti-rabbit IgG (H + L) Alexa Fluor 488 (dilution, 1:400; Thermo Fisher Scientific, Waltham, MA, USA) for 2 h at room temperature. Immunostaining was developed with a Histofine Simple Stain PO (M) Kit (Nichirei, Tokyo, Japan) per the instruction manual. Immunofluorescent images were obtained by a FluoView 1000 confocal microscope (Olympus, Tokyo, Japan) and a BZ-9000 microscope (Keyence, Osaka, Japan). The fluorescein intensities were calculated using ImageJ software.

### 4.10. Statistical Analysis

Data were expressed as the mean ± standard error of the mean. The changes in RBF were calculated as percentage changes from baseline. The n value indicates the number of animals studied. The Kolmogorov-Smirnov test was used to assess the normality of data distribution. Differences among groups and time points were assessed using a one-way or two-way repeated-measures ANOVA, as appropriate, followed by Dunnett’s test or the Holm-Sidak test. Prism 9 (GraphPad Software, San Diego, CA, USA) was used to perform all statistical analyses. *p*-values of <0.05 were considered statistically significant.

## Figures and Tables

**Table 1:** Longitudinal changes in casual blood glucose (mg/dL).

Age (Weeks)	6	8	10	12	14
db/db + normal feed (*n* = 6)	340.0 ± 41.8	419.0 ± 41.3	351.8 ± 19.8	331.5 ± 13.8	440.2 ± 54.0
db/db + Tofogliflozine (*n* = 6)	310.7 ± 30.0	226.0 ± 19.1 *	232.0 ± 32.4 *	202.5 ± 20.5 *	264.2 ± 43.3 *

Data are expressed as the mean ± standard error of the mean (SEM). * *p* < 0.05 vs. db/db + normal feed. These agents are commonly used by young obese patients with type 2 diabetes because weight reduction can be expected as calories are lost when glucose is excreted in the urine [3].

## Data Availability

The data presented in this study are available on request from the corresponding author.

## Supplementary Materials

Supplementary materials can be found at https://www.mdpi.com/article/10.3390/ijms23031362/s1.
