# Supplementary figures and images for: The Effect of Sodium-Dependent Glucose Cotransporter 2 Inhibitor Tofogliflozin on Neurovascular Coupling in the Retina in Type 2 Diabetic Mice
Source: Int J Mol Sci. 2022 Jan 25;23(3):1362. doi: 10.3390/ijms23031362 (PMC8835894; doi:10.3390/ijms23031362)

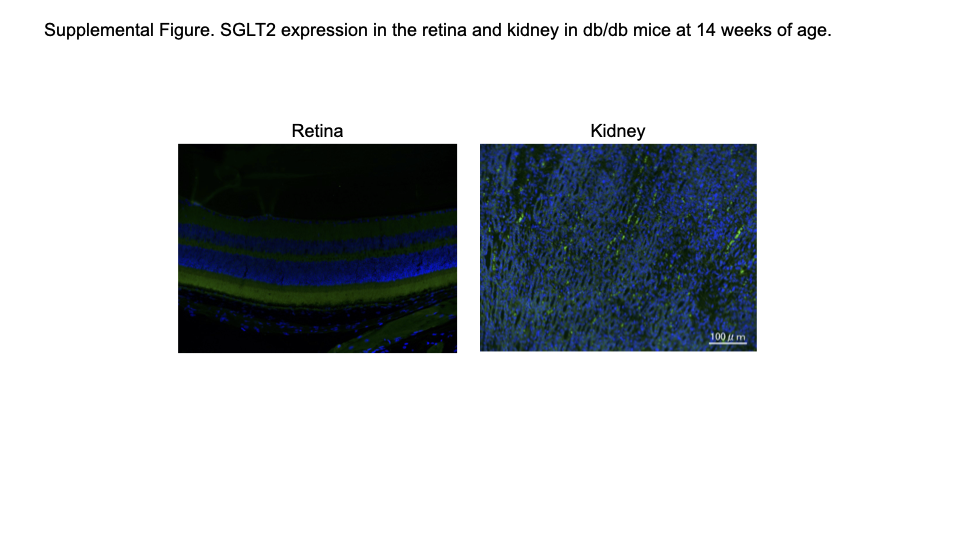

Supplement: Supplementary file 1 [file ijms-23-01362-s001.zip › ijms-1532313-supplementary.tiff]
